# Supplementary material for: Abortion as empowerment: reproductive rights activism in a legally restricted context
Source: BMC Pregnancy Childbirth. 2017 Nov 8;17(Suppl 2):350. doi: 10.1186/s12884-017-1498-y (PMC5688482; doi:10.1186/s12884-017-1498-y)
Supplement: Supplementary file 1 — Translation of 2-page clinic intake form. (PDF 34 kb) [file 12884_2017_1498_MOESM1_ESM.pdf]

(Name of clinic)

Health services for women requiring abortion care
Medical History

Date: _____/______/_________
Full Name: ______________________________
Age: ______________________ Government ID Number: ______________
Address: ______________________________
Telephone number: ______________________________
Referred by: ______________________________
Insurance Plan: ______________________
Nationality: ______________________
Education level: ______________________________
Occupation: ______________________________
Employment situation: ______________________________
Romantic relationship: ______________________________

Description of the situation: ______________________________
____
____

Has already made the decision: ___________ Alone: _________ Accompanied by: ____________
Has not made a decision: ______________________________

Clinical History

Sonogram	Date:	Date:	Date:	Date:

Date of last menstrual period (LMP):

Gestational age based on LMP ____________________ on ___________________________

Obstetric History
Pregnancies: ___________________________ Children (ages): ________________________
Births: ___________________________________
C-sections: _________ Date of last C-section:____________
Pregnancies not taken to term: _______________ Miscarriages: ________________
Induced abortions: _____________________ Method used to induce abortion: _____________
Medical practice used, or Alternative and Complementary Medicine: ____________________

Risk factors:

Blood type and RH factor: ____
Long term steroid treatment: ____________________________________
Severe anemia: ____
Blood clotting disorders: ____
Coronary or cardiovascular risk: __________________________________
Last C-section: ____
IUD: ____
Frequent aspirin use: ____

Useful information: ____
____
____
____
____
____

Interviews: ____
____
____
____
____
____
____
____
