# Supplementary material for: Abortion as empowerment: reproductive rights activism in a legally restricted context
Source: BMC Pregnancy Childbirth. 2017 Nov 8;17(Suppl 2):350. doi: 10.1186/s12884-017-1498-y (PMC5688482; doi:10.1186/s12884-017-1498-y)
Supplement: Supplementary file 2 — Translation of 3-page clinic intake form. (PDF 97 kb) [file 12884_2017_1498_MOESM2_ESM.pdf]

Instrument for evaluating the risk to the health of the woman due to pregnancy

Below are enumerated a series of situations which current Argentine law (article 86 of the national penal code, subsections 1 and 2) considers reasons for abortion that is not punishable by law.

Please check all of the situations which apply to you and explain:

- ☐ **The pregnancy puts my life at risk.**
- ☐ **The pregnancy is the result of a rape (sexual relationship that was not consensual).**
- ☐ The woman had sexual relations against her will: compelled physically, under coercion, or under force of threats.
- ☐ The woman did not consent to the conditions of contraception under which the sexual relationship took place.

The pregnancy puts my physical health at risk because I have:

- ☐ Conditions that are susceptible to worsening, or that are in fact worse as a result of the physiological changes produced by a normal pregnancy (anemia, chronic hypertension, rheumatoid conditions, orthopedic problems, blood clotting disorders, immunosuppressant conditions, diabetes, cardiopathy, uterine fibroids, varicose veins, epilepsy, psychiatric conditions, etc.) **INDIRECT HEALTH RISKS**
- ☐ Risk of medical complications of the pregnancy that can cause illness or worsen a preexisting illness or may threaten health or well-being (anemia, chronic hypertension, preeclampsia, obstetric hemorrhage, surgical risk, thrombosis, etc.) **DIRECT HEALTH RISKS**
- ☐ Illnesses that cannot be treated adequately during a pregnancy and that consequently increase health risks for women.
- ☐ Prior history of obstetrical complications. (Hemorrhage, uterine atony, preeclampsia, eclampsia, placental illnesses, sepsis, history of dangerous abortions/miscarriages, etc.)
- ☐ There are threats to physical integrity due to interpersonal violence.
- ☐ The woman's health is affected by a diagnosis of fetal deformity.

Socio-familial determinants that together with the pregnancy constitute a risk for the health of the woman (dimensions):

Employment:

- ☐ Impossible to continue in current employment because of the pregnancy.
- ☐ Unemployed, the pregnancy will not permit the patient to secure employment.
- ☐ Informal employment, impossible to take the leave necessary to safeguard her own health and the health of an infant.
- ☐ Economic dependence. [Patient] depends on others for her subsistence, and these individuals are not able to offer financial support for the pregnancy or future offspring.
- ☐ No income, low or insufficient to economically support a new member of the family.

Education

- ☐ Education in progress, which would have to be discontinued or delayed because of the pregnancy, with resulting postponement of employment and income.
- ☐ Low level of education, with related limits for accessing a decent job.
- ☐ Need to interrupt schooling because of the pregnancy.

Housing situation

Precarious housing because of:

- ☐ Poor conditions of current housing

- [ ] Difficulty paying rent, and constant risk of homelessness for self and future offspring.

- [ ] Living as a dependent, with resulting uncertainty about having a place to live and raise a child.

- [ ] Living with other families permanently or temporarily and unable to add another member to the family group.

Socio-familial conditions for raising a child:

- [ ] Lacking a partner or father to raise the child.

- [ ] Lacking support from a partner or father to raise the child.

- [ ] Lack of extended family network for raising a child.

- [ ] Number of dependent children and resulting impossibility of caring for an additional child.

- [ ] Health problems of children or other dependent family members, that occupy a lot of daily energy and would not allow [the patient] to care for a new family member.

- [ ] Unable to care for a child or an additional child because of work, including unpaid work and caring for the home.

- [ ] Imposed pregnancy resulting from negligence on the part of the health system that denied or delayed access to contraceptives.

Gender violence

- [ ] Woman who is a victim of gender violence by her partner, ex-partner, or others.

- [ ] The pregnancy would worsen the domestic violence that the woman is a victim of.

The pregnancy is a mental health risk because:

- [ ] The undesired pregnancy produces psychological suffering that affects the [patient's] wellbeing.

- [ ] Undergoing a traumatic situation (grief, post traumatic stress, sudden crisis)

- [ ] Currently experiencing puerperium, and the unplanned pregnancy cannot be undertaken because [patient] is currently caring for an infant.

- [ ] Psychological distress due to the diagnosis of an illness.

- [ ] There is psychological distress because of a diagnosis of fetal deformity.

- [ ] This pregnancy is early or late (relative to the woman's life cycle) which results in psychological distress.

If there are other issues that have not been mentioned, please write them below:

____
____
____
____
____
____
____

ASSESSMENT OF PSYCHOLOGICAL DISTRESS
PSYCHO-EMOTIONAL STATE OF THE PATIENT UPON RECEIVING NEWS OF THE PREGNANCY

Emotional Sphere	Somatic Sphere	Relational Sphere	Cognitive Sphere
☐ Unaltered	☐ Unaltered	☐ Unaltered	☐ Unaltered
☐ Calm	☐ Pain/physical malaise	☐ Aggressive	☐ Scattered
☐ Anguish	☐ Cutting/Self-harm	☐ Irritable	☐ Frequent negative thoughts
☐ Sad	☐ Sleep disturbances	☐ Isolated	☐ Self-reproach/Guilt
☐ Confused	☐ Change in appetite	☐ Inhibited	☐ Difficulty resolving problems
☐ Disoriented	☐ Substance abuse	☐ Paralyzed	☐ Difficulty making decisions
☐ Anxious	☐ Weakness/listlessness/fatigue	☐ Feeling alone	☐ Difficulty concentrating
☐ Exhausted/Tired	☐ Dizziness	☐ Difficulty undertaking daily activities	☐ Feelings of persecution/hallucinations
☐ Fearful	☐ Hyperactivity	☐ Other ____________________<br>__________________________	☐ Thinking of self-harm
☐ Hostile	☐ Shortness of breath		☐ Thinking of death
☐ Desperate	☐ Palpitations		☐ Other ____________________<br>__________________________
☐ Flat affect	☐ Other ____________________<br>__________________________		
☐ Hopeless			
☐ Impotent/overwhelmed			
☐ Feeling useless			
☐ Other ____________________<br>__________________________			

☐ The unwanted pregnancy produces psychological suffering that could trigger acts of self-harm.

☐ The forcible continuation of the pregnancy creates a risk of triggering psychological damage or pathology.

☐ There is a history of serious or chronic disorders (mood disorders, depressive disorders, anxiety disorders, depersonalization disorder).

☐ There is a history of severe post-partum mental health impairment.

☐ There is an intellectual disability.

☐ Future risk of adverse mental health outcome.
